# Supplementary material for: A Live Attenuated H1N1 Influenza Vaccine Based on the Mutated M Gene
Source: Vaccines (Basel). 2024 Jun 29;12(7):725. doi: 10.3390/vaccines12070725 (PMC11281364; doi:10.3390/vaccines12070725)
Supplement: Supplementary file 1 [file vaccines-12-00725-s001.zip › Figure legend of Supplementary Figure S1.pdf]

Supplementary Figure S1. Antibody responses in mice induced by intranasal administration of various doses of mPR8 vaccine. Formalin-inactivated whole PR8 virus vaccine was coated in 96-well ELISA plate. Then the serum sample to be tested was performed 2-times serial dilution firstly and then added on the coated plate. Biotinylated goat anti-mouse IgG antibody (Southern Biotechnology Associates, Inc. USA) was added. Then streptavidin-conjugated alkaline phosphatase (Southern Biotechnology Associates, Inc. USA) was added. After termination of the reaction, the OD450 absorbance was read using a SpectraMax M2e multifunction microplate reader (Molecular Devices). BALB/c mice were intranasally (i.n) immunized with different dose of mPR8 virus as indicated. The mice in control group were inoculated with the same volume of PBS, 3 mice per group. 21 days after immunization, the serum and nasal lavage fluid were collected for virus specific IgG(A) and IgA(B) assay respectively by ELISA. IgG1(C) and IgG(2a) in serum of the immunized mice were also determined by ELISA.
