# Supplementary figures and images for: A Live Attenuated H1N1 Influenza Vaccine Based on the Mutated M Gene
Source: Vaccines (Basel). 2024 Jun 29;12(7):725. doi: 10.3390/vaccines12070725 (PMC11281364; doi:10.3390/vaccines12070725)

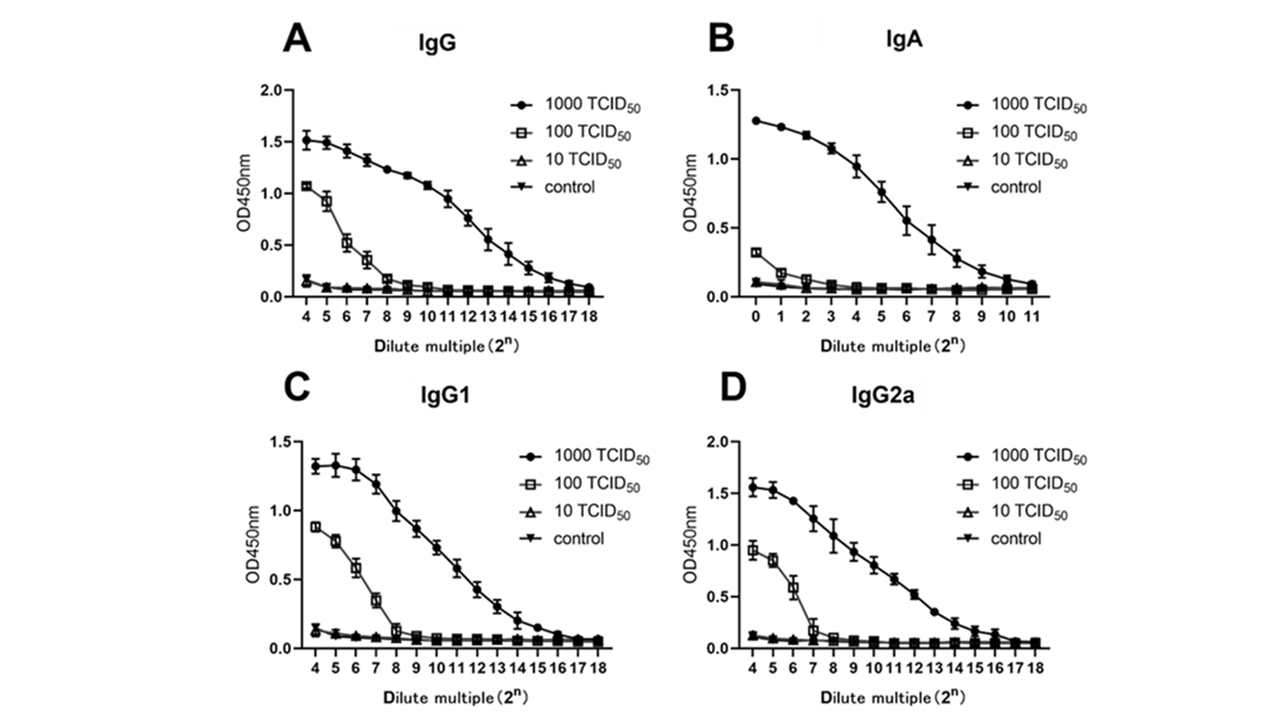

Supplement: Supplementary file 1 [file vaccines-12-00725-s001.zip › vaccines-3050272-Figure S1.tif]
